# Mitochondrial genome variation and intergenomic sequence transfers in *Hevea* species

**DOI:** 10.3389/fpls.2024.1234643

**Published:** 2024-04-10

**Authors:** Yingfeng Niu, Chengwen Gao, Jin Liu

**Affiliations:** ^1^ Yunnan Institute of Tropical Crops, National Key Laboratory for Biological Breeding of Tropical Crops, Yunnan Key Laboratory of Sustainable Utilization Research on Rubber Tree, Xishuangbanna, China; ^2^ Medical Research Center, The Affiliated Hospital of Qingdao University, Qingdao, China

**Keywords:** *Hevea*, mitochondrial genome, intergenomic sequence transfers, phylogenetic analysis, genome variation

## Abstract

Among the *Hevea* species, rubber tree (*Hevea brasiliensis*) is the most important source of natural rubber. In previous studies, we sequenced the complete nuclear and chloroplast genomes of *Hevea* species, providing an invaluable resource for studying their phylogeny, disease resistance, and breeding. However, given that plant mitochondrial genomes are more complex and more difficult to assemble than that of the other organelles, little is known about their mitochondrial genome, which limits the comprehensive understanding of *Hevea* genomic evolution. In this study, we sequenced and assembled the mitochondrial genomes of four *Hevea* species. The four mitochondrial genomes had consistent GC contents, codon usages and AT skews. However, there were significant differences in the genome lengths and sequence repeats. Specifically, the circular mitochondrial genomes of the four *Hevea* species ranged from 935,732 to 1,402,206 bp, with 34–35 unique protein-coding genes, 35–38 tRNA genes, and 6–13 rRNA genes. In addition, there were 17,294–46,552 bp intergenomic transfer fragments between the chloroplast and mitochondrial genomes, consisting of eight intact genes (*psaA, rrn16S, tRNA-Val, rrn5S, rrn4.5S, tRNA-Arg, tRNA-Asp*, and *tRNA-Asn*), intergenic spacer regions and partial gene sequences. The evolutionary position of *Hevea* species, crucial for understanding its adaptive strategies and relation to other species, was verified by phylogenetic analysis based on the protein-coding genes in the mitochondrial genomes of 21 Malpighiales species. The findings from this study not only provide valuable insights into the structure and evolution of the *Hevea* mitochondrial genome but also lay the foundation for further molecular, evolutionary studies, and genomic breeding studies on rubber tree and other *Hevea* species, thereby potentially informing conservation and utilization strategies.

## Introduction

Among the species in the genus *Hevea*, rubber tree (*Hevea brasiliensis*) is the most important source of natural rubber, accounting for over 98% of the global total natural rubber production (Cheng et al., 2023). The genus *Hevea* comprises ten species, including *H. spruceana, H. benthamiana, H. pauciflora, H. camargoana, H. brasiliensis, H. guianensis, H. microphylla, H. rigidifolia, H. nitida*, and *H. camporum*, exclusively distributed in the Amazon basin (Priyadarshan and Goncalves, 2003). There is no biological barrier on reproduction among these species, making it possible for inter-species crossing by artificial pollination (Priyadarshan and Clement-Demange, 2004). As a result, *Hevea* species are considered a species complex, forming a gene pool for breeding, especially in identifying and introducing genes encoding resistance against leaf diseases (Priyadarshan, 2003).

Mitochondria are essential for several metabolic processes, including adenosine triphosphate (ATP) synthesis and cellular respiration (Meyer et al., 2019). With the popularity and rapid development of sequencing technology, increased number of plant mitochondrial genomes has been assembled. However, out of the 12,029 terrestrial plant organelle genomes assembled to date (April 2023) based on the GenBank Organelle Genome Resources, only 477 are mitochondrial genomes and the rest are chloroplast genomes. This suggests that the mitochondrial genome is more complex and more difficult to assemble than that of the other organelles (Ma et al., 2022).

Mitochondrial genomes vary widely among plant species in terms of their length, gene content, and gene order (Richardson et al., 2013; Bi et al., 2020; Lai et al., 2022). In addition, the evolution of mitochondrial genomes has been characterized by many intergenomic sequence transfers and structural rearrangements (Wu et al.; Allen, 2015; Gao et al., 2023). At the same time, mitochondrial protein-coding genes are conserved, hence a potential for addressing unresolved phylogenetic questions (Tian et al., 2006; Hao et al., 2022). With the continuous development of sequencing technology and assembly methods (He et al., 2022), more and more plant mitochondrial genomes have been sequenced. In previous studies, we sequenced the complete nuclear genome of rubber tree (Liu et al., 2020). The chloroplast genomes of four species of the genus *Hevea* were also sequenced (Tangphatsornruang et al., 2011; Feng et al., 2020; Niu et al., 2020a, b; Hu et al., 2022). But only one mitochondrial genome of *Hevea* species has been released, which needs further exploration (Shearman et al., 2014). The study on the mitochondrial genome of *Hevea* species will lay a foundation for further evolutionary and genomic breeding studies on rubber tree. In this study, we newly determined four *Hevea* mitochondrial genome sequences and performed comparative genomic analysis. This study aimed to: (1) perform a comparative analysis of the mitochondrial genome features of *Hevea* species; (2) identify the intergenomic sequence transfers between mitochondrial and chloroplast genomes in *Hevea* species; (3) reconstruct the *Hevea* species phylogeny using the mitochondrial gene sequences. This study will provide a better understanding of the interspecific differences in the genus *Hevea* and will be valuable for further research on genomic breeding studies on rubber tree.

## Materials and methods

### Samples collection and DNA sequencing

We collected fresh leaf samples of four *Hevea* species (*H. benthamiana*, *H. camargoana*, *H. pauciflora*, and *H. spruceana*) from the rubber tree germplasm resource nursery of the Chinese Academy of Tropical Agriculture Science (N 19°34′31.53″and E 109°31′17.97″). The total genomic DNA from the leaves was extracted using the CTAB method (Li et al., 2013). The genomic DNA was used for library construction. For next-generation sequencing, libraries with insert sizes of 350 bp were constructed and sequenced using the Illumina Novaseq6000 platform (Illumina, USA). For third-generation sequencing, 15 Kb fragment libraries were constructed by the PacBio RS II platform (Pacific Biosciences, Menlo Park, CA, USA). A total of 10.2–16.6 GB of raw sequencing data, of which the third-generation sequencing raw data was 5.5–10.1 GB (**Supplementary Figure S1**), and a mitochondrial genome depth of coverage of 138.5–489.8× was obtained from the four *Hevea* species.

### Mitochondrial genome assembly and annotation

The short reads were filtered using Trimmomatic v0.38 (Bolger et al., 2014), and their *de novo* assembly was performed using SPAdes v3.5.0 with different K-mer parameters (Bankevich et al., 2012). GSAT software (He et al., 2022) was used to calculate the mitochondrial genome graph-based framework, the genomic structure maps of *H. camargoana* and *H. spruceana* mitochondria shows complex relationships between genomic sequences (**Supplementary Figure S2**). The obtained PacBio reads were *de novo* assembled using canu v2.2 and PAMT (Koren et al., 2017; Bi et al., 2024) in hifi mode, with a genome size set to 2M, and default parameters for the rest, and the draft assemblies were polished by Pilon (Walker et al., 2014). During the running of Pilon, only paired-mapped reads were used to correct, which can eliminate nuclear mitochondrial DNA segments (NUMT) as much as possible. A total of 14–110 contigs were obtained by canu assembler, the longest was 309,883 bp in length, and the average length was 53,687 bp. The circularization process consists of the following steps: A. The establishment of coherent linkages among segments, B. Extensive and uniform coverage at the sequence assembly junctions, C. The wholeness of gene segments along with consistent and meaningful coding sequences, and D. An appropriate correlation between the repetition rates in repetitive segments and the depth of sequencing, ultimately leading to the effective and comprehensive assembly of the entire genome. The assembly process was summarized in **Supplementary Figure S3**. At the same time, we calculated base depth and corrected the mitochondrial sequence using genotypes that matched the sequencing depth of mitochondrial sequence. The IGV software to analyze the reasonableness of sequencing read coverage and to determine if there were complete third-generation sequencing reads spanning the low-depth regions. For regions with low sequencing coverage or complex structures, validation was performed with Sanger sequencing to ensure the circular genomic sequence was accurate and complete. Specifically, the Primer Premier software was used to design the primers for each low sequencing coverage or complex structures region. The genomic DNA was used as the template to amplify the target DNA fragment. After purification and identification, PCR amplification and sequencing was performed. Subsequently, the mitochondrial genome was annotated by GeSeq (Tillich et al., 2017) and ORF Finder (https://www.ncbi.nlm.nih.gov/orffinder/) using the *Hevea brasiliensis* (AP014526) mitochondrial genome as the reference genome. After GeSeq annotation, the BLASTN and BLASTP alignment tools were used to identify and compare the mitochondrial sequences (Zhang et al., 2000), and the annotation results were manually modified. In addition, the tRNAs were annotated using tRNAscan-SE 2.0 (Lowe and Chan, 2016) and ARWEN (Laslett and Canback, 2008). Finally, the circular mitochondrial genome of *Hevea* species was mapped using Proksee (https://proksee.ca/).

### Codon usage and repetitive sequences analysis

Relative synonymous codon usage values were analyzed using MEGA v7.0.26 (Kumar et al., 2016). Codon usage heatmap was plotted using the R package pheatmap v1.0.12. Next, the AT and GC skews were calculated using the formulas: AT skew = (A − T)/(A + T), and GC skew = (G − C)/(G + C), respectively. In addition, the long repeats in the mitochondrial genome were analyzed using REPuter (Kurtz et al., 2001). Finally, the simple sequence repeats (SSRs) were identified using the MISA v2.1 software (Beier et al., 2017), with 10, 6, 5, 5, 5, and 5 as the minimum numbers of repeats for mono-, di-, tri-, tetra-, penta-, and hexa-nucleotides, respectively. Tandem repeats were identified using the program Tandem Repeats Finder (TRF) (Benson, 1999).

We selected the *H. benthamiana* mitochondrial genome, which has the highest similarity to the rubber tree mitochondrial genome, for paired repetitive sequence detection using BLASTN, and to identify potential recombinations from these repetitive sequences. We selected two of them for PCR validation. The primers at the ends of repeats were designed by Primer Premier3 v4.1 (https://bioinfo.ut.ee/primer3-0.4.0/) (**Supplementary Table S12**). The PCR amplification system was 40 µL in total, consisting of 1 µL of template gDNA, 2 µL of forward and reserve primer, 20 µL of 2× Rapid Taq Master Mix, and 15 µL of ddH_2_O. The PCR amplification program was 94 °C denaturation for 3 min, 35 cycles of 94 °C for 30 s, 56 °C for 30 s, and 72 °C for 60 s, and a 72 °C final extension for 5 min.

### Intergenomic sequence transfer analysis

The mitochondrial genomes of the four *Hevea* species were searched by BLASTN against the chloroplast genomes (Zhang et al., 2000), using parameters “-evalue 1e-6 -outfmt 6”, and length > 100 bp to identify transfer fragments between the two genomes. The Intergenomic sequence transfer fragments between chloroplast and mitochondrial genomes were then mapped using Circos v0.69 software (Krzywinski et al., 2009).

### Identification of RNA editing sites

The Deepred-mt (Edera et al., 2021) was used for the prediction of RNA editing sites of the mitochondrial genomes. It predicts C-to-U editing events based on the 40 nucleotides flanking a given cytidine.

### Phylogenetic analysis

Phylogenetic analysis was performed for 21 Malpighiales species using *Passiflora edulis* as the outgroup. The mitochondrial genomes of the 21 Malpighiales species, including the four *Hevea* species were downloaded from the Genbank. Next, the mitochondrial protein-coding genes (*atp1, atp4, atp6, atp8, atp9, ccmB, ccmC, ccmFc, ccmFn, cox1, cox2, cox3, cob, matR, nad7, nad9, nd1, nd2, nd3, nd4, nd4L, nd5, nd6, prl10, prl16, rps1, rps12, rps3*, and *rps4*) common to all the species were extracted to construct a phylogenetic tree. The mitochondrial gene sequences of Malpighiales species were aligned using Muscle v.3.8.31 (Edgar, 2004). A phylogenetic tree was built with a maximum likelihood (ML) approach using RAxML v8.1.5 (Stamatakis, 2006) based on the best-fit substitution model (GTR+I+G) determined by jModelTest v2.1.7 (Posada, 2008). Branch support was assessed using bootstrap resampling with 1000 replicates.

## Results

### General features of the *Hevea* mitochondrial genomes

**Figure 1:**
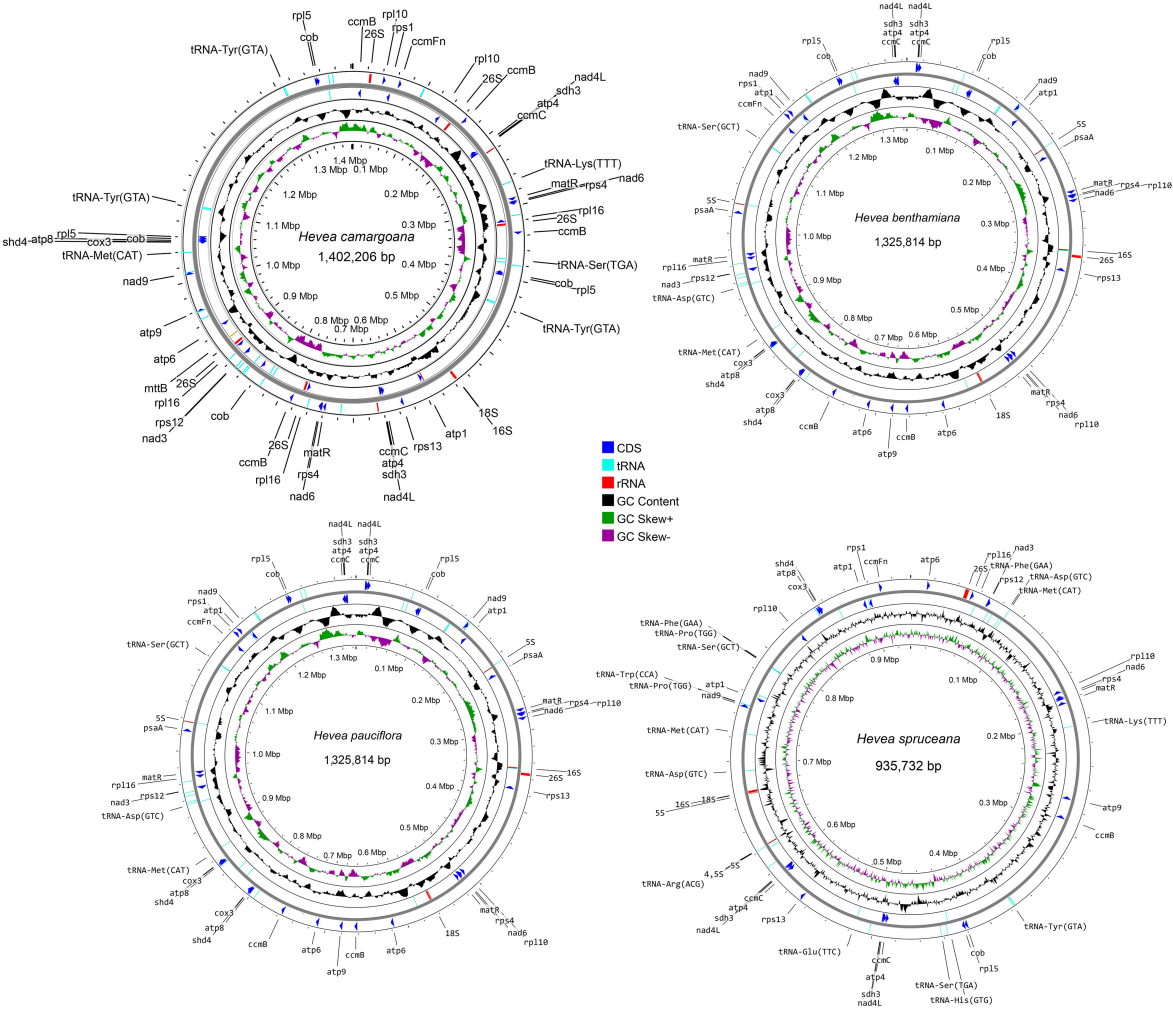
The mitochondrial genomes of the four *Hevea species*. The gene map, GC content, and GC skew are presented from the outside to the inside of the mitochondrial genome map, respectively.

Raw mitochondrial genome sequence data were obtained from *H. benthamiana* (OR663908), *H. camargoana* (OR663909), *H. pauciflora* (OR663910), and *H. spruceana* (OR663911) and were successfully assembled as a single circular genome. The sizes of the four newly sequenced *Hevea* mitochondrial genomes ranged from 935,732 to 1,402,206 bp (**Figure 1**; **Table 1**), with GC contents of 44.17–44.24%. Based on the comparative analysis, the four *Hevea* mitochondrial genomes had 34–35 unique protein-coding genes, 35–38 tRNA genes, and 6–13 rRNA genes (**Supplementary Tables S1–S4**). Notably, the number of *H. spruceana* mitochondrial genes was less than that of the other three species, mainly because the copy number of some genes of *H. spruceana* (*ccmB, cob*, and *matR* et al.) was only a single copy (**Supplementary Figure S4**; **Supplementary Table S5**). The four newly sequenced *Hevea* mitochondrial genomes have been submitted to the GenBank with accession numbers OR663908–OR663911.

**Table 1:** Features of *Hevea* mitochondrial genomes.

Species Name	Genome Size	Unique protein-coding genes	rRNA	tRNA	GC (%)	Accession no.
*H. benthamiana*	1,325,814	35	8	38	44.17	OR663908
*H. camargoana*	1,402,206	34	13	36	44.13	OR663909
*H. pauciflora*	1,325,814	35	8	38	44.17	OR663910
*H. spruceana*	935,732	35	6	35	44.24	OR663911
*H. brasiliensis*	1,325,823	36	8	38	44.17	AP014526

### Intergenomic sequence transfers between mitochondrial and chloroplast genomes

**Figure 2:**
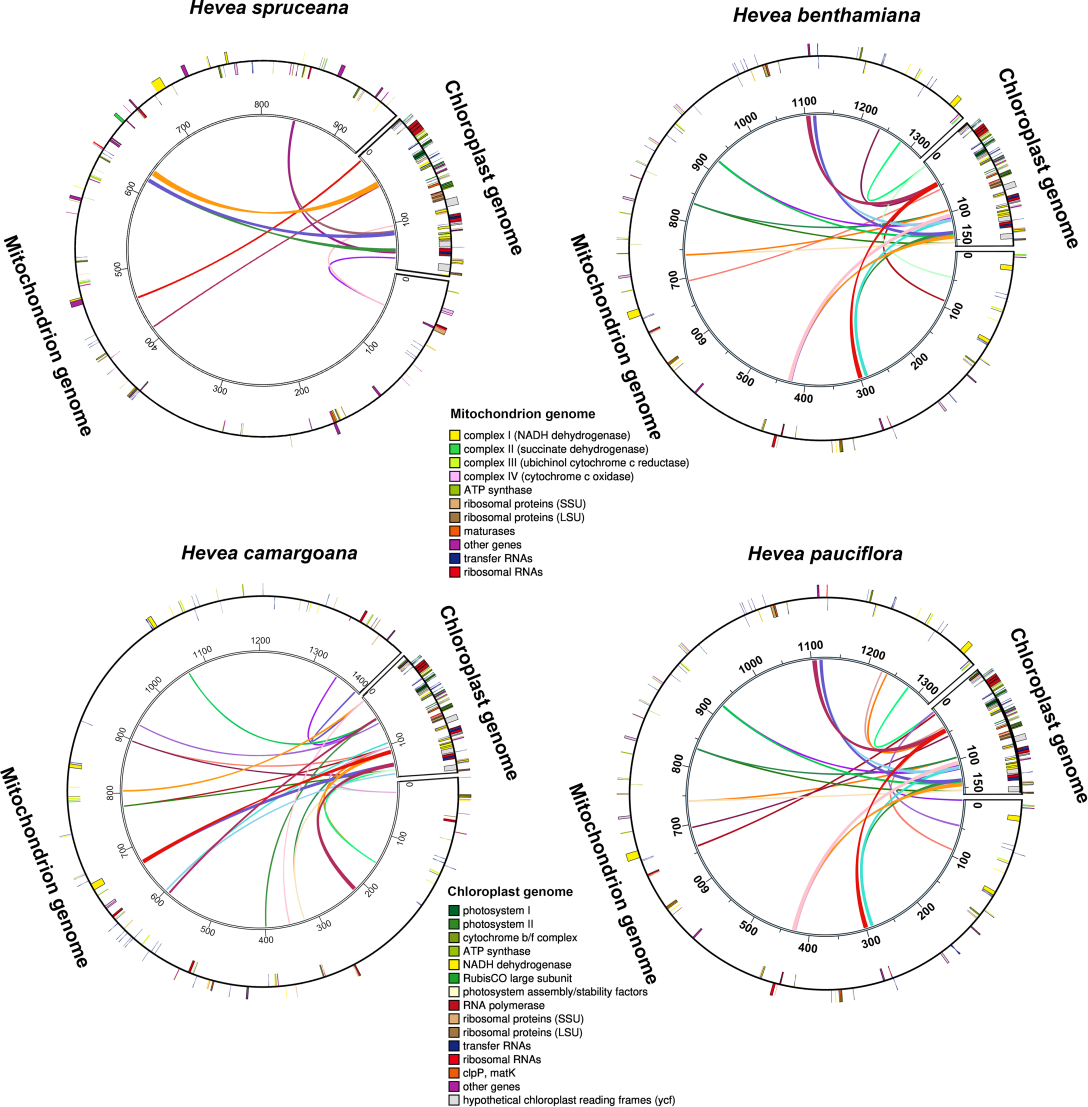
Schematic diagram of sequence transfer between mitochondrial and chloroplast genomes in four *Hevea species*. Colored lines within the circles represents intergenomic transfer sequences; genes shown outside and inside the circle were transcribed in a counterclockwise and clockwise manner, respectively.

The length of the *Hevea* mitochondrial genomes was approximately 5.8–8.7 times that of chloroplast genomes (MT333859, MN781109, NC_059798, and NC_059799). There were 17,294–46,552 bp long common sequence fragments between the chloroplast and mitochondrial genomes in the four *Hevea* species, including intergenic and gene regions (**Figure 2**; **Supplementary Table S6**), which accounted for 1.9–3.5% of the mitochondrial genome and 10.7–28.9% of the chloroplast genome. The transfer sequences from the chloroplast to the mitochondrial genomes in the four *Hevea* species contained eight intact genes (*psaA, rrn16S, tRNA-Val, rrn5S, rrn4.5S, tRNA-Arg, tRNA-Asp*, and *tRNA-Asn*), intergenic spacers regions and partial gene sequences (*psaB, ycf3, tRNA-Ile, ycf1, rrn23S, ycf1, tRNA-Ala, ycf2, atpE*, *ndhF*, and *rps16*). Most of these transfer sequences are located in the IR region (50/79) of the chloroplast genomes (**Supplementary Table S6**).

### Protein-coding genes and codon usages

**Figure 3:**
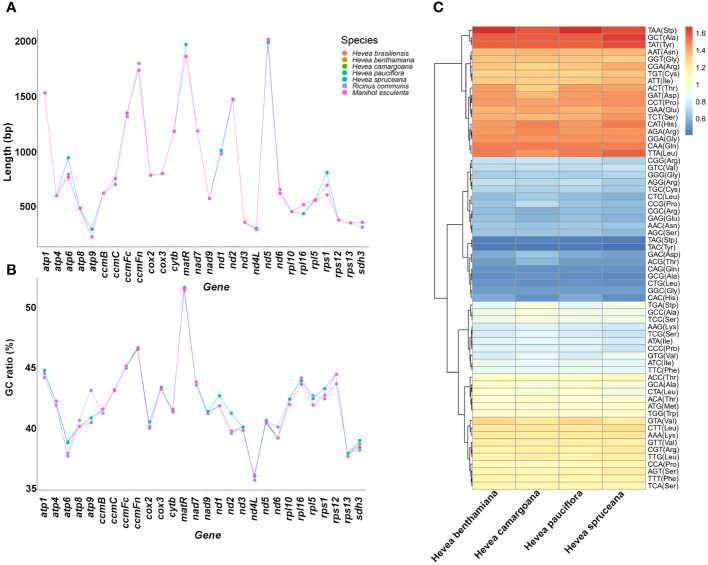
Protein-coding genes and the relative synonymous codon occurrence in *Hevea* mitochondrial genomes. **(A)** The length of the protein-coding genes. **(B)** The GC ratio of the protein-coding genes. **(C)** The relative synonymous codon occurrence in the protein-coding genes.

The length of most *Hevea* mitochondrial protein-coding genes (PCGs) is conserved (**Figure 3A**; **Supplementary Tables S1–S4**). In addition, the majority strand PCGs had a negative GC and positive AT skews, while the minority strand PCGs had positive GC and negative AT skews. Notably, the PCGs GC contents and AT skews were consistent across the *Hevea* mitochondrial genomes (**Figure 3B**; **Supplementary Table S7**).

The relative synonymous codon usage analysis of the mitochondrial genome revealed that among the four *Hevea* species, there were 64 codons encoding 20 amino acids (**Figure 3C**; **Supplementary Figure S5**; **Supplementary Table S8**). The total number of codons ranged from 12,292 to 18,395, with TTT as the most frequent (456–661 instances) codon, and TGC as the least (55–87 instances) among the PCGs in the *Hevea* mitochondrial genomes. The codon usage pattern was largely consistent in the four *Hevea* mitochondrial genomes.

### Analysis of repeats in the *Hevea* mitochondrial genomes

Analysis of the *Hevea* mitochondrial genome repeats revealed 453–677 long repeats with a total length of 322,471–555,902 bp, accounting for 23.0–59.4% of the mitochondrial genomes (**Supplementary Table S9**). Most of the long repeats were 30 to 50 bp in length, and the longest was 116,525 bp in *H. benthamiana*.

Tandem repeats, consisting of core repeat units are repeated many times in a tandem pattern. We detected a total of 61–93 tandem repeats in the mitochondrial genome of *Hevea* mitochondrial genomes with lengths between 5 and 76 bp (**Supplementary Table S10**).

In addition, 98–133 SSRs were identified in *Hevea* mitochondrial genomes, with a total size of 1,306 to 2,030 bp (**Supplementary Table S11**). Mononucleotide repeats were the most common, followed by di-, tri- and hexa- repeats, respectively. Most SSRs were located in the intergenic or intronic regions, except for 1–2 SSRs that were in the *nad1* protein-coding region.

There were two repeat sequences that could potentially mediate the homologous recombination in *H. benthamiana* mitochondrial genome. We performed PCR amplification and Sanger sequencing to verify the presence of the minor conformation. The scheme of the primer design was shown in **Supplementary Figure S6A** and **Supplementary Table S12**. Both sets of specific primers were successful in amplifying fragments of the repeat sequence fragments. Upon interchanging the reverse primers, we observed that they retained the ability to amplify these repeat sequence fragments, as shown in **Supplementary Figure S6B**. Based on these validation outcomes, we can infer the potential types of homologous recombination in the *H. benthamiana* mitochondrial genome, illustrated in **Supplementary Figure S6A**. Both conformations A and B are loop structures, each spanning a total length of 1,325,823 base pairs, and are differentiated by the orientation of their intermediate sequences, which are either forward or reverse. Due to the presence of direct repeats, conformation C consists of loops that form a two-chromosomal structure.

### Identification of RNA editing sites

We predicted a total of 436–443 potential C-to-U RNA editing sites in *Hevea* mitochondrial PCGs (**Supplementary Table S13**; **Supplementary Figure S7**). The predicted RNA editing sites of the mitochondrial genes of the four *Hevea* species are shown in **Supplementary Figure S6**. Among these mitochondrial PCGs, we identified 37 RNA editing sites in *nad4* and 35 for *ccmB* gene, which were the most frequent among these PCGs. In contrast, the gene *ycf3* had only 0–1 C-to-U editing sites in *Hevea* mitochondrial genes.

### Phylogenetic analysis based on mitochondrial genome sequences

**Figure 4:**
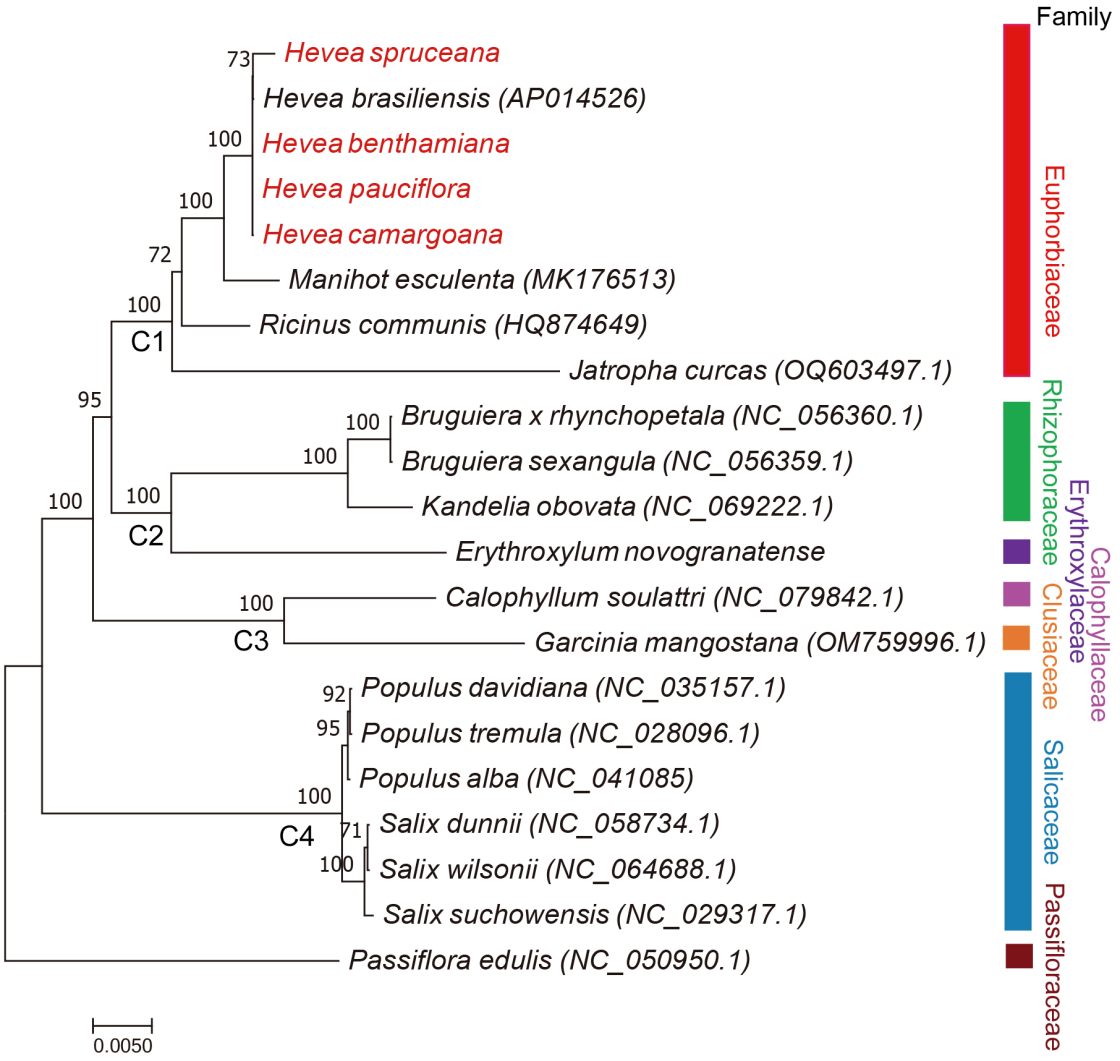
ML phylogenetic tree of Malpighiales species based on the common mitochondrial protein-coding genes. The numbers indicate the maximum likelihood bootstrap values (BS = 100% were not showed).

Phylogenetic analysis was performed based on 29 common mitochondrial genes, from 21 Malpighiales species. Phylogenetic analysis revealed that most branches of the phylogenetic tree had high ML bootstraps values. Four well-supported clades (C1, C2, C3, and C4 Clade) were recovered within Malpighiale. C1 Clade included family Euphorbiaceae. C2 Clade included families Rhizophoraceae and Erythroxylaceae. The Calophyllaceae and Clusiaceae families formed C3 Clade. C4 Clade included family Salicaceae. Salicaceae species formed a monophyletic clade and diverged earlier. The five *Hevea* species (Euphorbiaceae) were clustered together (**Figure 4**).

## Discussion

The mitochondrial genomes of terrestrial plants have undergone dramatic structural changes that have complicated their genomic composition, making plant mitochondrial genome research relatively challenging (Morley and Nielsen, 2017; Kozik et al., 2019; Wu et al., 2022). Notably, the mitochondrial genome size varies considerably among different plant species (Wynn and Christensen, 2019; Jackman et al., 2020), with the mitochondrial genomes of *Hevea* species being nearly twice the Euphorbiaceae species (Rivarola et al., 2011; Tao et al., 2019). In this study, we sequenced and assembled the mitochondrial genomes of four *Hevea* species. Their mitochondrial genomes ranged from 935,732 bp (*H. spruceana*) to 1,402,206 bp (*H. camargoana*). However, their GC content was the same. Similar to the mitochondrial genomes of most plants, most sequences in the *Hevea* mitochondrial genomes are non-genes, with gene sequences ranging from 4.87% (*H. spruceana*) to 5.23% (*H. camargoana*) of the total mitochondrial genome, possibly due to the gradual increase in sequence repeats during evolution (Ma et al., 2022). In addition, most PCGs in the *Hevea* mitochondrial genomes use the canonical ATG as the start codon, while *nad1* and *rps4* genes use ACG as the start codon, a phenomenon also reported in other studies and attributed to RNA editing modifications (Sloan et al., 2018; Xu et al., 2021).

Repetitive sequences, widely found in mitochondrial genomes, are important source of information for developing markers for evolutionary and population analysis (Nishikawa et al., 2005; Liu et al., 2015; Xiong et al., 2022). Herein, there were relatively large differences in the repetitive sequences in the *Hevea* mitochondrial genomes. Therefore, the four newly sequenced *Hevea* mitochondrial genomes provide new resources on the structure and function of the *Hevea* mitochondrial genomes. The *Hevea* mitochondrial genomes contains abundant repetitive sequences, which may indicate frequent intermolecular recombination in the *Hevea* mitochondrial genomes. The repetitive sequences identified in this study could mediate the formation of two conformations in the *H. benthamiana* mitochondrial genome (**Supplementary Figure S5**). The specific DNA repair of mitochondrial genome may be related to these phenomena (Liu et al., 2023; Yang et al., 2023). The results of this study support the existence of multiple conformations in plant mitochondrial genomes. Intergenomic sequence transfers from chloroplast to mitochondrial genome is common during terrestrial plant evolution (Gui et al., 2016; Nguyen et al., 2020). Consistent with the findings in other genera (Niu et al., 2021, 2022), the *Hevea* chloroplast sequences were inserted into the mitochondrial genome at different locations. The extensive rearrangement observed within the mitochondrial genome may be attributable to intergenomic sequence transfers. This hypothesis is supported by the observation that segments of the chloroplast genome which have migrated into the mitochondria exhibit a high degree of alignment with the original chloroplast genome sequences. Furthermore, the insertion locations of these segments appear to be randomly distributed. In the *Hevea* mitochondrial genome, the cumulative length of these transferred fragments spans from 17,294 to 46,552 bp, a range significantly exceeding that of transfer fragments identified in other genera (Gao et al., 2023). The transferred fragments and repetitive sequences are likely responsible for the size difference among the *Hevea* mitochondrial genomes. The fragments transferred from the *Hevea* chloroplast to the mitochondrial genome included four tRNA, three rRNA, and *psaA*, tRNA genes, which is also a common phenomenon in angiosperms (Bi et al., 2016; Cheng et al., 2021). In addition, these transferred fragments contain partial chloroplast gene sequences that have no function in the mitochondrial genome, which may be due to the loss of function of some genes during sequence transfer. This phenomenon suggests that the sequence transfer was from the chloroplast into the mitochondrial genome (Hao and Palmer, 2009; Smith, 2011).

The genetic relationship among *Hevea* species has been revealed through the analysis of nuclear genes, internal ribosome transcription spacer and chloroplast genes (Cheng et al., 2009; Tangphatsornruang et al., 2011; Zhai et al., 2014). However, research on the reconstruction plant phylogeny of mitochondrial genome sequences is scarce. In this study, a phylogenetic tree was constructed using PCGs in the mitochondrial genome to explore the evolutionary relationships among Malpighiales species. The phylogenetic topology of the mitochondrial phylogenetic tree was generally consistent with previous studies (Xi et al., 2012; Endress et al., 2013). The findings from this study complement the genetic knowledge on genus *Hevea*, and provide a new perspective to study the evolution of *Hevea* species.

### Conclusions

The *Hevea* mitochondrial genomes have consistent GC contents, codon occurrence and AT skews. However, there are significant differences in their lengths and sequence repeats. In addition, there are 17,294–46,552 bp intergenomic transfer fragments between the *Hevea* mitochondrial and chloroplast genomes. The evolutionary position of *Hevea* species was verified by phylogenetic analysis based on PCGs of the mitochondrial genome of 21 Malpighiales species. The findings from this study provide valuable genetic resources for further studies on *Hevea* species.

## Collection of plant material

We have permission to collect *Hevea* species. The collection of plant material comply with relevant institutional, national, and international guidelines and legislation.

## Data availability statement

The datasets presented in this study can be found in online repositories. The names of the repository/repositories and accession number(s) can be found below: GenBank on the NCBI website at https://www.ncbi.nlm.nih.gov, using the accession numbers OQ658721–OQ658724, and accession numbers OR663908–OR663911, the raw sequencing data have been deposited at the NCBI Sequence Read Archive (SRA) under accession PRJNA946487.

## Author contributions

YN and JL conceived the study, wrote and revised the manuscript. CG performed the data analyses and drafted the manuscript. All authors read and approved the final manuscript.
