# Supplementary figures and images for: Mitochondrial genome variation and intergenomic sequence transfers in Hevea species
Source: Front Plant Sci. 2024 Apr 10;15:1234643. doi: 10.3389/fpls.2024.1234643 (PMC11039855; doi:10.3389/fpls.2024.1234643)

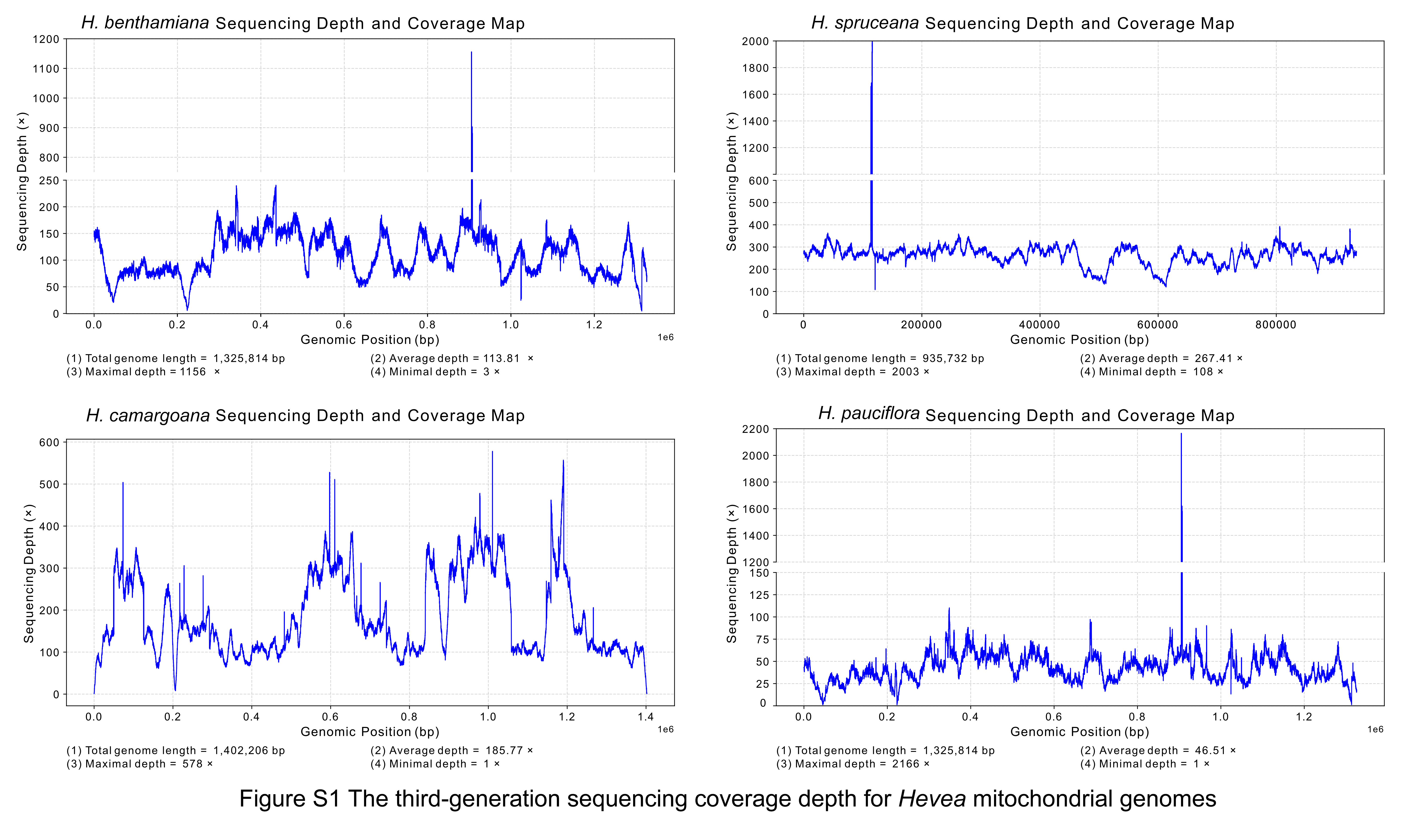

Supplement: Supplementary file 1 [file DataSheet_1.zip › Figure S1.JPEG]

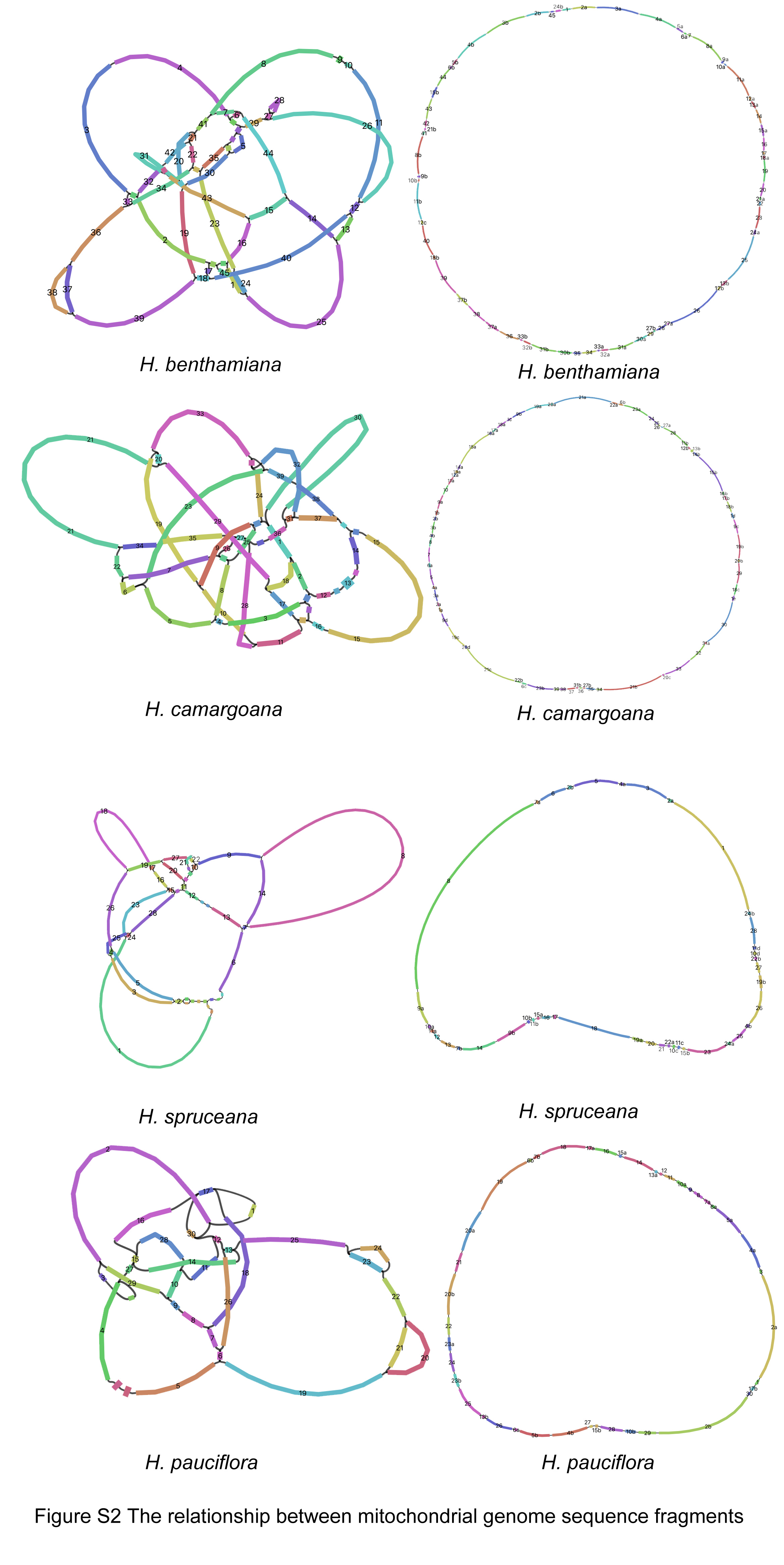

Supplement: Supplementary file 1 [file DataSheet_1.zip › Figure S2.JPEG]

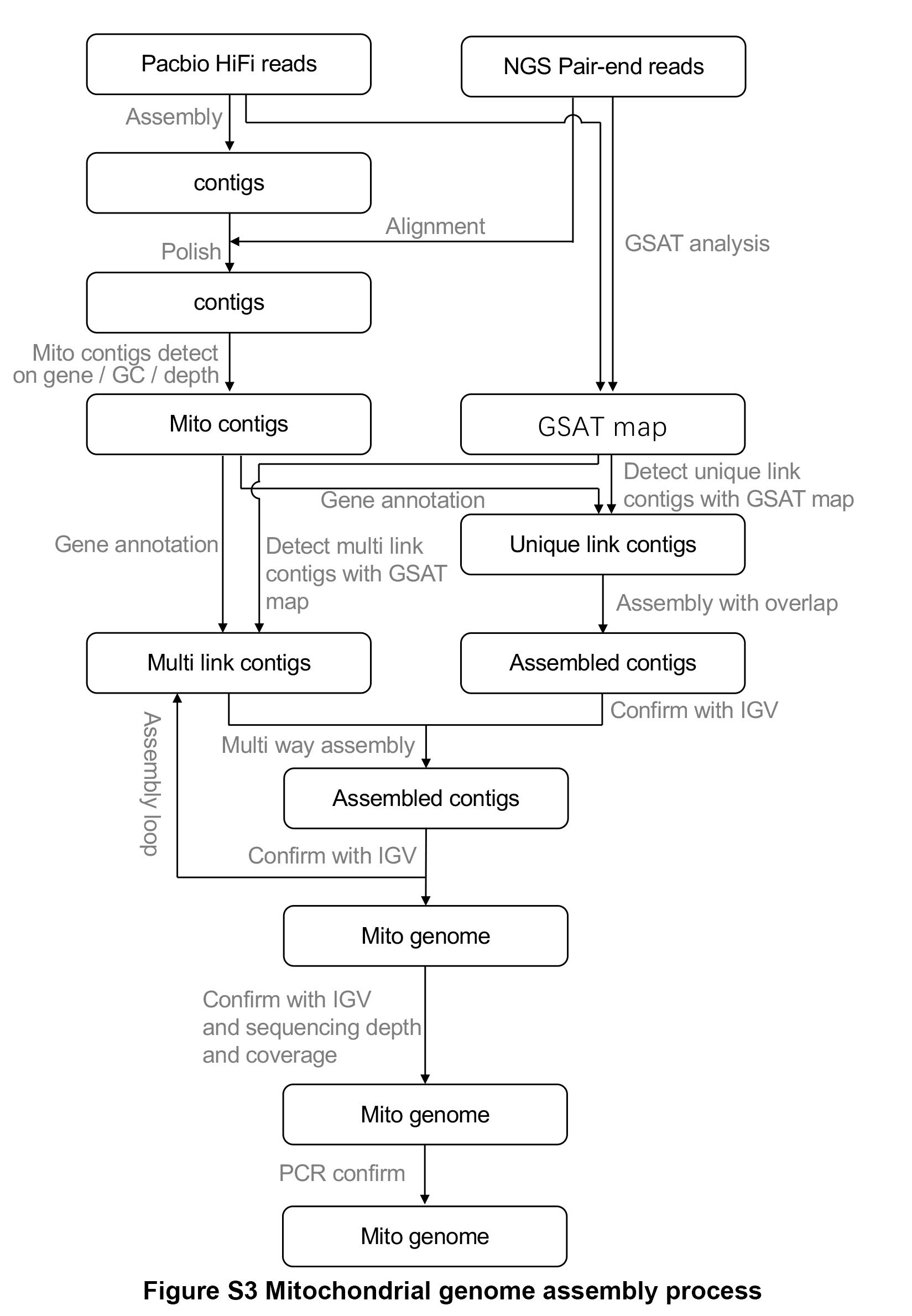

Supplement: Supplementary file 1 [file DataSheet_1.zip › Figure S3.JPEG]

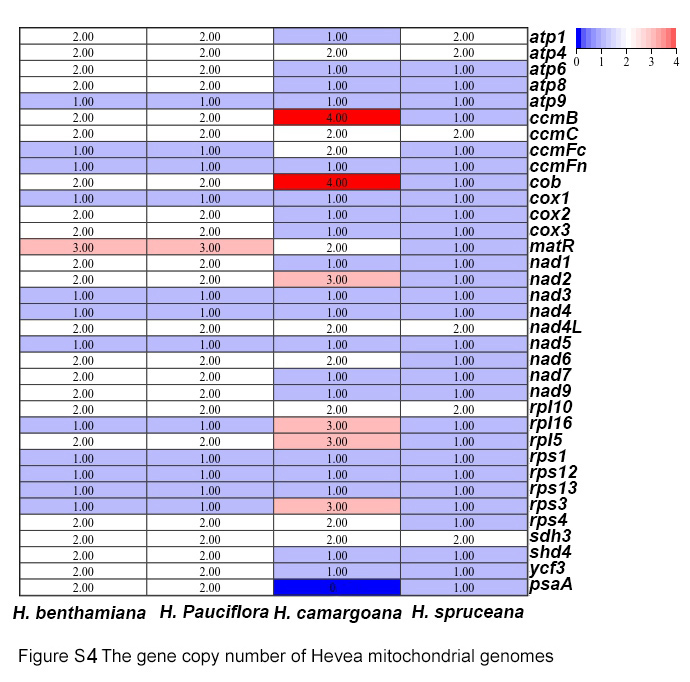

Supplement: Supplementary file 1 [file DataSheet_1.zip › Figure S4.JPEG]

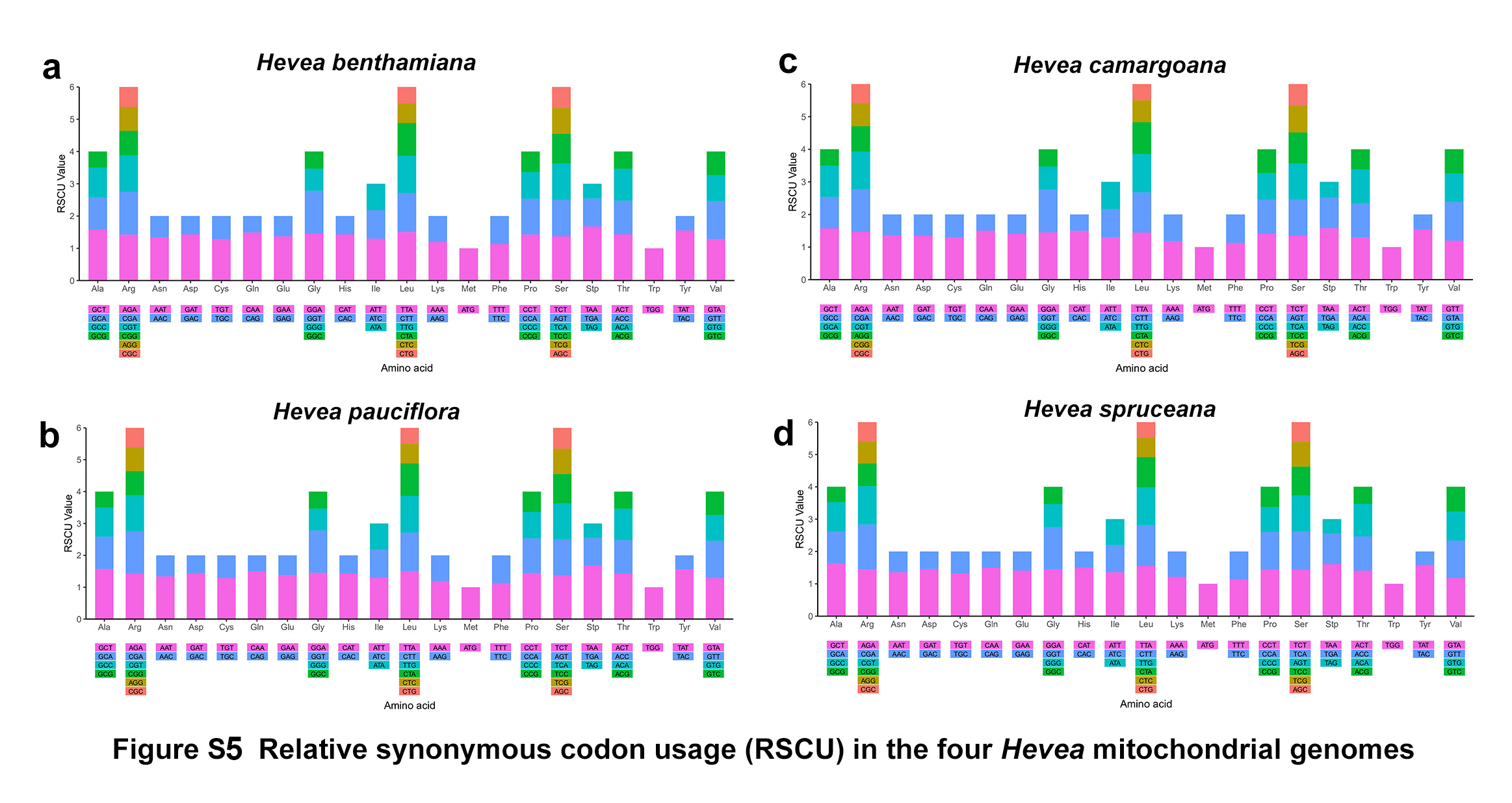

Supplement: Supplementary file 1 [file DataSheet_1.zip › Figure S5.JPEG]

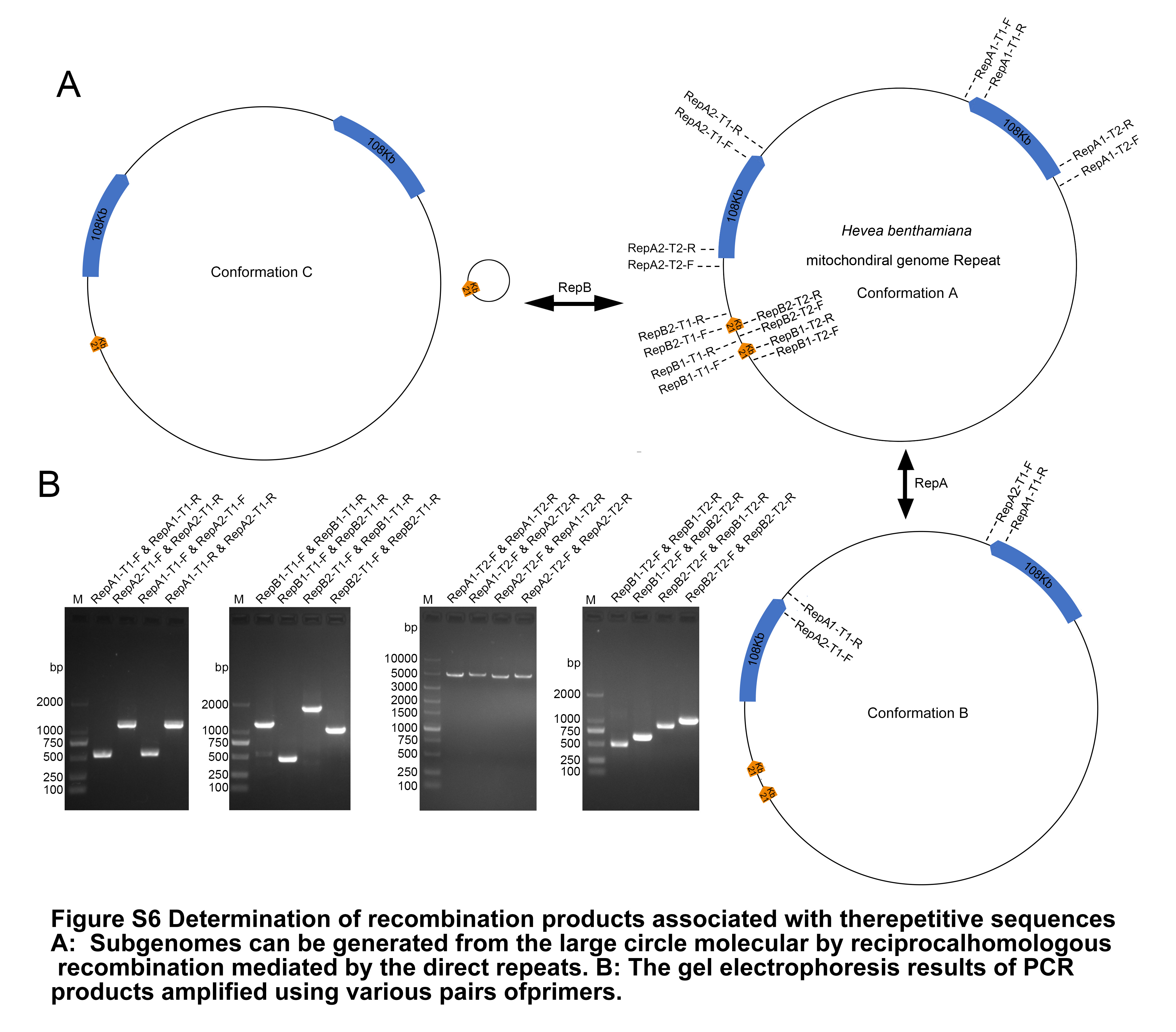

Supplement: Supplementary file 1 [file DataSheet_1.zip › Figure S6.JPEG]

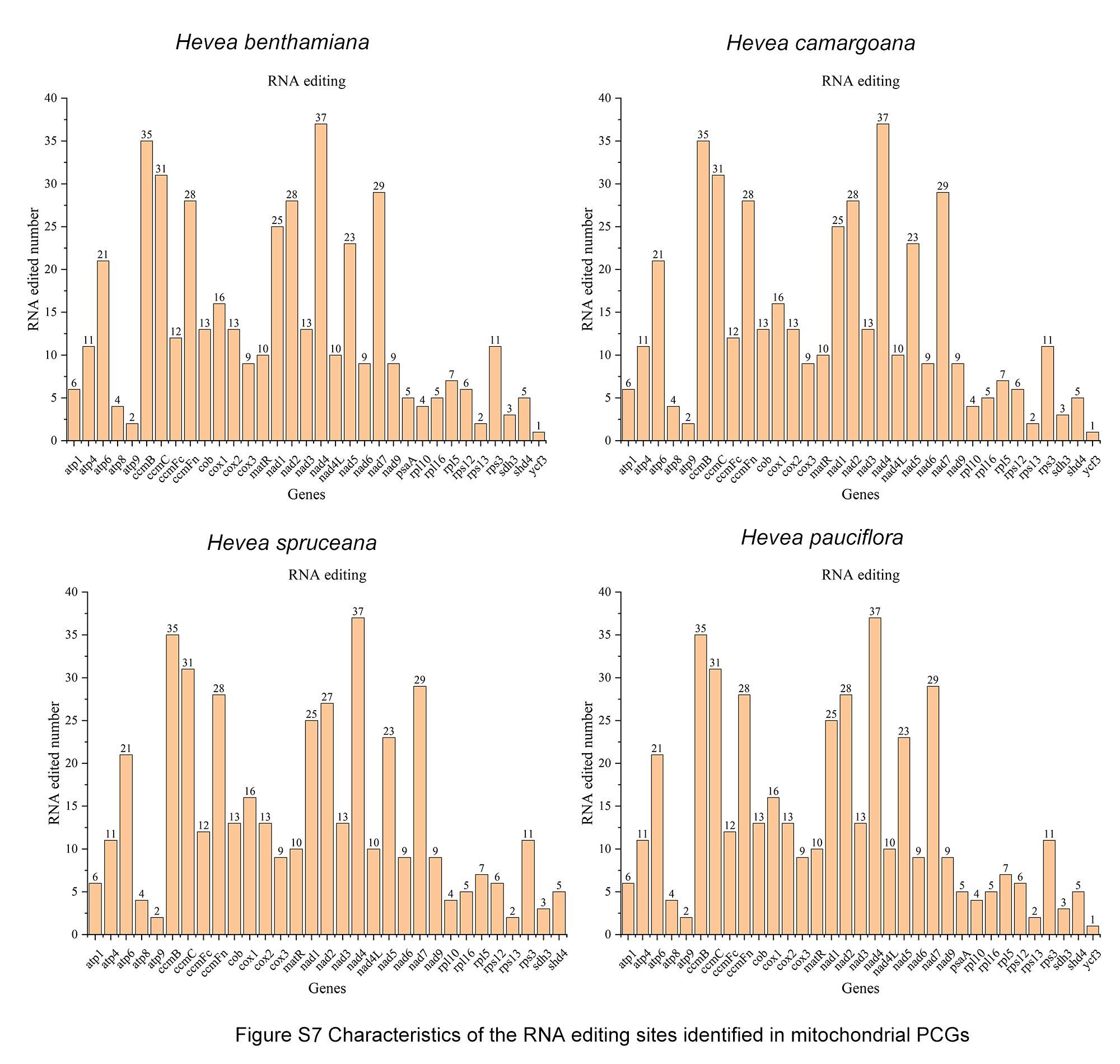

Supplement: Supplementary file 1 [file DataSheet_1.zip › Figure S7.JPEG]
